# Longitudinal, prospective study of head impacts in male high school football players

**DOI:** 10.1371/journal.pone.0291374

**Published:** 2023-09-08

**Authors:** Kelsey L. McAlister, Wendy J. Mack, Cynthia Bir, David A. Baron, Christine Som, Karen Li, Anthony Chavarria-Garcia, Siddhant Sawardekar, David Baron, Zachary Toth, Courtney Allem, Nicholas Beatty, Junko Nakayama, Ryan Kelln, Tracy Zaslow, Ravi Bansal, Bradley S. Peterson

**Affiliations:** 1 Department of Population and Public Health Sciences, Keck School of Medicine, University of Southern California, Los Angeles, CA, United States of America; 2 Department of Biomedical Engineering, Wayne State University, Detroit, MI, United States of America; 3 Western University, Pomona, CA, United States of America; 4 Institute for the Developing Mind, Children’s Hospital, Los Angeles, CA, United States of America; 5 Crescenta Valley High School, La Crescenta, CA, United States of America; 6 Department of Pediatrics, Keck School of Medicine, University of Southern California, Los Angeles, CA, United States of America; 7 Department of Psychiatry, Keck School of Medicine, University of Southern California, Los Angeles, CA, United States of America; Opole University of Technology: Politechnika Opolska, POLAND

## Abstract

**Introduction:**

Repetitive, subconcussive events may adversely affect the brain and cognition during sensitive periods of development. Prevention of neurocognitive consequences of concussion in high school football is therefore an important public health priority. We aimed to identify the player positions and demographic, behavioral, cognitive, and impact characteristics that predict the frequency and acceleration of head impacts in high school football players.

**Methods:**

In this prospective study, three cohorts of adolescent male athletes (N = 53, 28.3% Hispanic) were recruited over three successive seasons in a high school American football program. Demographic and cognitive functioning were assessed at baseline prior to participating in football. Helmet sensors recorded impact frequency and acceleration. Each head impact was captured on film from five different angles. Research staff verified and characterized on-field impacts. Player-level Poisson regressions and year-level and impact-level linear mixed-effect models were used to determine demographic, behavioral, cognitive, and impact characteristics as predictors of impact frequency and acceleration.

**Results:**

4,678 valid impacts were recorded. Impact frequency positively associated with baseline symptoms of hyperactivity-impulsivity [β(SE) = 1.05 impacts per year per unit of symptom severity (1.00), p = 0.01] and inattentiveness [β(SE) = 1.003 impacts per year per T-score unit (1.001), p = 0.01]. Compared to quarterbacks, the highest acceleration impacts were sustained by kickers/punters [β(SE) = 21.5 g’s higher (7.1), p = 0.002], kick/punt returners [β(SE) = 9.3 g’s higher (4.4), p = 0.03], and defensive backs [β(SE) = 4.9 g’s higher (2.5), p = 0.05]. Impacts were more frequent in the second [β(SE) = 33.4 impacts (14.2), p = 0.02)] and third [β(SE) = 50.9 impacts (20.1), p = 0.01] year of play. Acceleration was highest in top-of-the-head impacts [β(SE) = 4.4 g’s higher (0.8), p<0.001].

**Conclusion:**

Including screening questions for Attention-Deficit/Hyperactivity Disorder in pre-participation evaluations can help identify a subset of prospective football players who may be at risk for increased head impacts. Position-specific strategies to modify kickoffs and correct tackling and blocking may also reduce impact burden.

## Introduction

Sport-related head injuries are highly prevalent in youth [1], and young compared with adult athletes are more susceptible to concussion [2–4]. Adults typically require 10–14 days for clinical recovery from concussion [3, 5], whereas adolescents require 2–4 weeks or more [6]. Sport-related concussions in youth are generally thought to have adverse long-term effects on a variety of health outcomes, including: changes in brain structure and function; poorer memory, executive functioning, and motor control; more frequent mood disorders; and poorer academic performance [7]. The risk for sport-related concussion has been shown to vary with personal characteristics, including age, race, ethnicity, prior mental health problems, and prior history of concussions [8–10], suggesting the possibility of identifying youth who may benefit from specific strategies to mitigate the risk for sport-related concussions.

Among all sports for US youth, football is the leading source of concussions, owing to the large number and excessive acceleration of head impacts sustained [11]. Moreover, subconcussive head impacts (blows to the head that do not cause clinically detectable symptoms) occur frequently and are of increasing concern in American football, because recent studies have shown that they can disrupt brain structure and function, and impair cognition [12–16]. Brain imaging studies, for example, have reported disturbances in the white matter of male high school football players after one year of play [17–20]. Other studies of high school football players have reported that a greater number of head impacts after a year of play is significantly associated with neurophysiological and cognitive impairments [13, 21], including poorer memory [18, 19]. These studies, however, did not assess whether cognitive impairments were present before beginning football or whether cognitive problems predispose subsequently to more frequent and high-force impacts. One retrospective study estimated that the odds of developing Chronic Traumatic Encephalopathy doubles every 2.6 years of football played [22], suggesting that enduring brain injury likely scales with the cumulative number of plays in which a football athlete participates over time.

Similar to sports-related head injuries more generally, player-specific characteristics may increase the risk and force of head impacts in American football. These include player position (e.g., quarterback, running back, defensive lineman) [23–28], play type (e.g., pass play, running play, special teams play) [25, 29], head impact location (e.g., front or side of the head) [24, 25, 28], and what the head strikes (e.g., the ground, another player’s helmet) [23, 25], though the evidence is inconsistent and the risk may vary across levels of competition (e.g., freshman or varsity team membership). Moreover, demographic characteristics have not been studied extensively as potential modifiers for the risk of head impacts or their adverse long-term health consequences. Nevertheless, studies of young athletes suggest that older players (10–13 years old) sustain a larger number and more forceful head impacts than younger players (9–11 years old) [30, 31]. A recent systematic review of studies for several contact sports (e.g., football, soccer, ice hockey) that used electronic sensors to detect and quantify head impacts in both males and females identified potential demographic risk factors for head impacts [32], including a positive association of impact force with age but not other demographic characteristics in football players [32]. Other football studies have not formally assessed the associations of demographic attributes with impact frequency and acceleration [32].

Prior studies of adolescent athletes in other sports suggest that the presence of Attention-Deficit/Hyperactivity Disorder (ADHD) is an additional personal characteristic that may confer an increased risk for more frequent and forceful head impacts in American football. For example, adolescent athletes in other sports who have ADHD have higher rates of concussion [33–35], consistent with the higher risk for injuries and acceidents that youth with ADHD have in the general population [36–38]. Major limitations of prior studies associating ADHD with concussion rates, however, were that the diagnosis of ADHD was self-reported [33–35], and only concussion frequency was assessed, not whether ADHD symptoms were associated with more frequent or more severe head impacts. In addition to conferring a greater risk for concussion, pre-injury mental health problems, including ADHD, have been associated with a longer duration of symptoms and prolonged recovery following a sport-related concussion [39]. This greater vulnerability of ADHD youth to the adverse consequences of head injury elevates the importance of discerning whether they are also at greater risk for injury from repeated subconcussive head impacts.

Thus, prior studies have yielded neither a clear nor a complete understanding of the possible risk factors for head impacts and their health consequences in high school football players. With more than one million high school students participating in football each year [40] and the increasing evidence for the adverse long-term consequences of subconcussive head impacts, understanding the player characteristics that predict of the number and cumulative force of subconcussive impacts is essential for developing specific coaching and officiating strategies, protective gear, game rules, and policies of youth athletic organizations that will protect young athletes and reduce their adverse long-term health consequences from repeated head impacts.

The aim of this study was to assess the associations of personal player characteristics prior to involvement in contact sports (their demographics, pre-existing emotional and behavioral symptoms, intelligence and other cognitive measures), player position (quarterback, lineman, offensive or defensive back, etc.), and impact characteristics (helmet-to-helmet, helmet-to-ground, etc.) with the frequency and acceleration of repeated head impacts in subsequent play. We hypothesized that (1) the frequency of impacts would associate significantly with pre-existing symptoms of ADHD, and (2) head acceleration would be greatest in helmet-to-helmet and helmet-to-ground impacts, and with playing on special teams.

## Methods

Data were drawn from an ongoing longitudinal study of brain development and mental health in adolescent football players. High school football players were recruited as incoming freshmen from Crescenta Valley High School in La Crescenta, CA—a large, suburban 4-year high school. Research staff attended athletic recruitment events and parent informational meetings. Informational flyers were provided at non-contact summer practices.

Youth were included if they were football players entering their freshman year and planned to attend Crescenta Valley High School for all 4 years. The sample was exclusively male, as no females participated on the team. Youth were excluded who 1) had IQ below 80, 2) had a history of seizures or neurological illness, 3) had sustained prior concussions by a child or parent report, 4) had any contraindication to magnetic resonance imaging, 5) had a history of contact sport or football participation, or were participating in other football leagues or contact sports during the duration of the study, by a child or parent report. Parents provided written informed consent and youth provided written assent. Youth received $200 as compensation for completing all procedures at each visit. The Institutional Review Board of Children’s Hospital Los Angeles approved this study.

Baseline visits occurred in the summer before full-contact football practices in the freshman year. Consent and assent were conducted at the baseline visit. Participants then underwent baseline assessments, including demographic and behavioral surveys, and neuropsychological testing. Following baseline assessments, participants then completed their first year of play. Annual assessments, including repeat behavioral surveys and neuropsychological testing, occurred in the summer prior to their next year of play. Head impact data were collected during all practices and games, but data only from games are reported here, as practices consistently and intentionally minimized contact to minimize player injuries.

**Fig 1:**
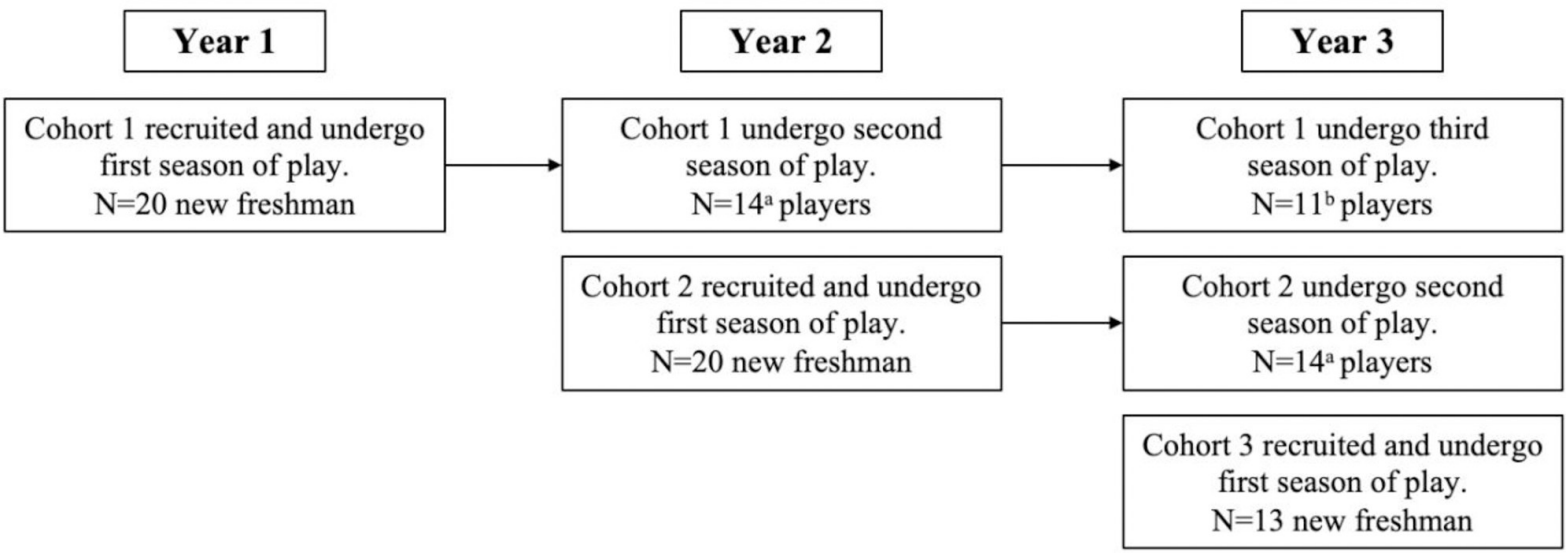
Study timeline outlining recruitment and continuation of football per each cohort. ^a^n = 6 discontinued football. ^b^n = 3 discontinued football.

From the summer of 2017 through the summer of 2019, we recruited three successive player cohorts. Three years of data were available (Fig 1). In Year 1 for Cohort 1, we recruited 20 high school football players entering their freshman year. Of these 20 players, 14 completed the first annual assessment and continued football in Year 2 (6 players dropped out before their second year); 11 completed the second annual assessment and continued football in Year 3 (3 players dropped out before their third year). In Year 2, for Cohort 2, we recruited 20 high school football players entering their freshman year. Of these 20 players, 14 completed the first annual assessment and continued football in Year 3 (6 players dropped out before their second year). In Year 3, for Cohort 3, we recruited 13 high school football players entering their freshman year.

### Measures

Demographics were obtained via questionnaire (Table 1). Physical measures included height and weight. To determine fitness level, youth performed 15 standard push-ups, measuring time for the heart and respiratory rate to return to baseline after exertion [41]. Intelligence was estimated using the Wechsler Abbreviated Scale of Intelligence-2^nd^ Edition [42]. Attention and inhibitory control were assessed using the Connors’ Continuous Performance Test (CPT)-Version 3.0 [43], yielding T-scores for detectability (discrimination between targets and non-targets), omissions (missed targets), commissions (incorrect responses to non-targets), and average response speed [43]. Parents reported symptoms of ADHD using the ADHD Rating Scale, yielding an overall ADHD symptom score and subscale scores for inattention and hyperactivity-impulsivity [44].

**Table 1:** Participant baseline demographic, behavioral, and cognitive characteristics (N = 53 players).

	All Cohorts (N = 53)	Cohort 1 (N = 20)	Cohort 2 (N = 20)	Cohort 3 (N = 13)
Demographic Variables	Mean ± SD	Mean ± SD	Mean ± SD	Mean ± SD
Age (in years)	14.4 ± 0.4	14.4 ± 0.4	14.3 ± 0.3	14.3 ± 0.4
Annual Household Income (in $100,000)	1.3 ± 0.1^a^	1.3 ± 0.1^b^	1.3 ± 0.1^d^	1.3 ± 0.1
Height (cm)	170.9 ± 7.7^c^	170.6 ± 7.1^d^	170.1 ± 7.9	172.5 ± 8.5
Weight (kg)	73.7 ± 19.5^c^	73.5 ± 20.9^d^	72.1 ± 18.4	76.4 ± 20.2
BMI (kg/m^2^)	25.1 ± 5.9^c^	25.2 ± 6.9^d^	24.8 ± 5.5	25.5 ± 5.7
Push-up test time to recovery (minutes)	1.7 ± 0.8^a^	1.6 ± 0.8^d^	1.3 ± 0.6^b^	2.5 ± 0.7
	**N(%)**	**N(%)**	**N(%)**	**N(%)**
Ethnicity				
Hispanic/Latino/Spanish origin	15 (28)	7 (35)	6 (30)	2 (15)
Not Hispanic/Latino/Spanish origin	36 (68)	13 (65)	13 (65)	10 (77)
Declined to state	2 (4)	0 (0)	1 (5)	1 (8)
Race				
White	37 (70)	13 (65)	14 (70)	10 (77)
Black/African American	3 (6)	0 (0)	2 (10)	1 (8)
Asian	3 (6)	2 (10)	1 (5)	0 (0)
Native Hawaiian or Other Pacific Islander	3 (6)	2 (10)	1 (5)	0 (0)
Other	3 (6)	1 (5)	1 (5)	1 (8)
More than one race	4 (8)	2 (10)	1 (5)	1 (8)
**Behavioral and Cognitive Variables**	**Mean ± SD**	**Mean ± SD**	**Mean ± SD**	**Mean ± SD**
WASI IQ score	103.0 ± 12.2	105.8 ± 13.8	101.7 ± 11.6	100.7 ± 10.4
ADHD symptom score	7.9 ± 9.2	8.5 ± 10.7	6.7 ± 5.8	9.0 ± 11.2
Inattention symptom score	5.8 ± 5.7	6.0 ± 6.3	5.0 ± 4.6	6.9 ± 6.7
Hyperactivity-impulsivity symptom score	2.1 ± 4.2	2.6 ± 4.8	1.7 ± 2.0	2.2 ± 5.7
Conners’ CPT				
Detectability T-score	57.4 ± 9.4	57.9 ± 8.6	54.0 ± 10.0	62.0 ± 7.9
Reaction time T-score	50.4 ± 8.6	50.6 ± 7.1	51.7 ± 9.4	48.1 ± 9.7
Omissions T-score	58.4 ± 15.4	57.0 ± 12.4	58.0 ± 18.1	61.2 ± 16.0
Commission T-score	54.6 ± 7.7	55.7 ± 7.6	50.9 ± 7.0	58.5 ± 6.5

^a^*n* = 50

^b^*n* = 18

^c^*n* = 52

^d^*n* = 19

Abbreviations: BMI: body mass index, WASI IQ: Wechsler Abbreviated Scale of Intelligence, ADHD: Attention-Deficit/Hyperactivity Disorder, CPT: Continuous Performance Test.

### Head impacts

Participants wore a Riddell football helmet (Riddell Inc, Chicago, IL). The Riddell InSite Impact Response System, which included a five-zone sensor pad [45], was used for Cohort 1 for the first seven games of Year 1. Research-based conversions developed by Riddell automatically classified location (top, front, back, left, right) and acceleration bin (low: 15–28.9g, medium: 29–62.9g, high: 63+g) tallied in 5-minute segments. Because the InSite System did not collect precise timestamps and continuous acceleration data, this was retired mid-season for Cohort 1. Thereafter, helmets were equipped with the Riddell Head Impact Telemetry System (HITS; Simbex, Lebanon, NH). The in-helmet sensor contained 6 non-orthogonally-mounted, single-axis accelerometers that recorded acceleration-time history at 1000 Hertz [46, 47]. Peak linear acceleration (g) and location were recorded for each impact, defined as acceleration >10 g’s; data from 8 milliseconds before and 32 milliseconds after impact were recorded. These limits were selected because they were recommended by Riddell’s technical team, and the limits have been employed in prior studies in male football players [24, 28, 48]. The HITS had a built-in algorithm (described elsewhere [49]) that excluded false impacts (i.e., a player dropping or throwing the helmet) that did not match the theoretical pattern for rigid body head acceleration. The two sensor systems had identical thresholds of 15g for recording an impact.

The Insite and the HITS sensors were pre-specified to send alerts to a sideline receiver unit for impacts exceeding 63 g because this value represents the top 1% of impacts reported in prior studies of known concussive events [45]. Upon an alert, the athletic trainer or medical staff would evaluate the player for concussion using the Sport Concussion Achievement Tool Version 5 (SCAT-5). Copies of the SCAT-5 were obtained by research staff. Research staff at the game recorded the time of the alert for later viewing on the video recordings.

The HITS system provided continuous acceleration in g’s and thereby provided more precise impact data than the Insite System, which provided acceleration as an ordinal variable. We therefore elected to conduct primary analyses using only data derived from HITS. Secondary analyses combining data from the Insite and HITS systems are presented in S1 File.

### Video capture

**Fig 2:**
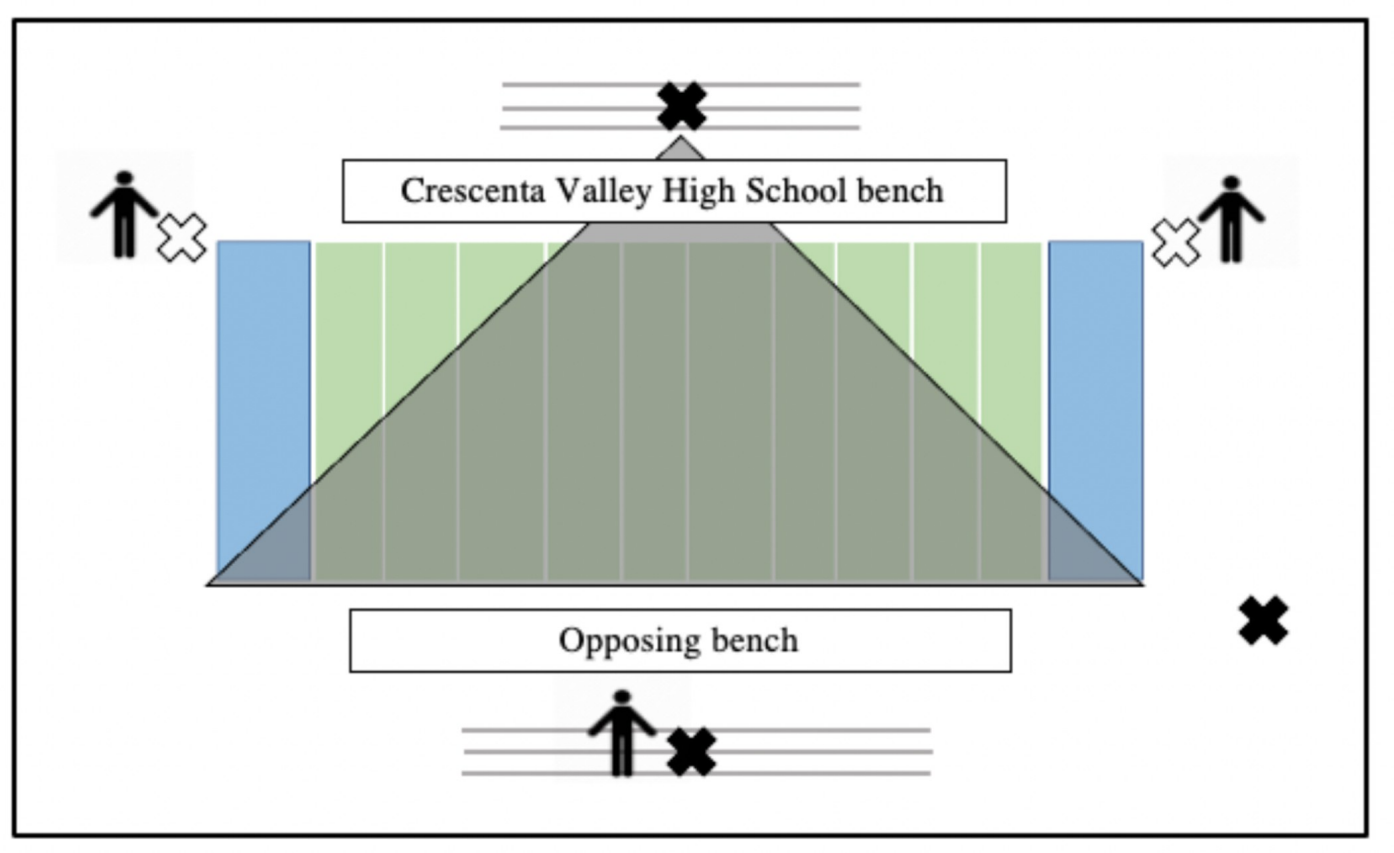
Diagram of the 5-camera set-up with the approximate angle of an elevated camera. White x = on-field camera. Black x = on-field camera. Human figure = student volunteer and/or research staff following the action.

Four or five video cameras (Sony Handy Cam FDR-AX33 4K; Sony Action Cam HDR-AS300R 60p) secured to tripods acquired video recordings of every game (Fig 2). Videos were time-stamped using a world clock on a cellular phone showing the hour, minutes, and seconds at the start of each recording, which allowed us to synchronize the time of each impact with the correct time on the video recording to verify and characterize true impacts. False impacts (impacts that did not occur as a result of an active play, such as a player dropping his helmet at half-time) were removed. Research staff recorded the following for each valid impact: external source of the impact (helmet, shoulder, torso, hand/elbow, or knee/leg/foot of another player; the ground or ball; impact from whiplash), impact sequence (i.e., where the impact fell in a sequence of impacts; primary, secondary, tertiary, quaternary), on-field team (offense, defense, special teams), and play type (pass, run, special teams). Player positions for each impact were categorized as center, offensive guard or tackle, tight end, wide receiver, running back, quarterback, defensive tackle or end, linebacker, defensive back, kicker/punter, kick-punt returner, and special teams. Number of years played was considered experience level, and team [i.e., freshman, junior varsity (JV), varsity] was treated as the assigned level of play. Impact characteristics were recorded as unclear if the characteristic was unviewable (e.g., players in a heap). Research staff tallied the total number of plays each participant was involved in and position during each play, regardless of impact occurrence, to permit calculation of impact risk per play and position. The total number of plays in which each player participated was summed for that player within each year of play (e.g. the total number of plays joined in Year 1); the average number of plays joined per number of years played was also calculated.

### Statistical analyses

Stata V.15 was used for all analyses. Univariate statistics were calculated for all baseline measures. Impact rates per play by position were generated by summing the total number of impacts sustained in each position and dividing by the total number of plays participated in corresponding position. Primary analyses were conducted using HITS-only data (i.e., continuous acceleration in g’s); secondary analyses combining data from the Insite and HITS (i.e., ordinal acceleration) systems are presented in S1 File. We conducted player-, year-, and impact-level analyses to address our aims.

### Sensitivity analyses

We used player-level analysis of variance (ANOVA) (for continuous characteristics) and Pearson χ^2^ (for categorical characteristcs) models to test differences in demographic, behavioral, and cognitive characteristics among players who discontinued football and players who continued. We used player-level ANOVA (for continuous characteristics) and Pearson χ^2^ (for categorical characteristcs) models to identify potential differences in demographic, behavioral, cognitive, and impact characteristics across the three cohorts.

### Univariate, player-level analyses

We used player-level univariate models to identify predictors of head impact frequency from demographic information and behavioral and cognitive characteristics present before starting football or any other contact sport. We used separate player-level Poisson regression and negative binomial models (when appropriate due to Poisson over-dispersion) to assess whether each baseline measure (independent variable) was associated with impact frequency per year (dependent variable), adjusting for the average number of plays joined per year. For each player, the total number of impacts sustained across all years of play was calculated and used as the outcome measure; the total number of years (i.e., seasons of football) played was used as the offset variable. The final results from the models then reflected the frequency of head impacts per year played. Because of the change of head impact sensors mid-season for Cohort 1, we used only head impact data from 4 of the 10 games played in the first year. We therefore reduced the years of play for Cohort 1 participants to indicate that data from their first year of play was obtained using a fraction of the year, rather than from an entire year, thereby adjusting for the missing HITS data from the first 6 games of Cohort 1 Year 1. This analysis used one observation per player.

### Multivariate, year-level analyses

We used year-level linear mixed-effect models to assess the association of age and year (independent variables) on impact frequency and acceleration (dependent variables), adjusted for total number of plays during the corresponding year; models for age additionally adjusted for number of years played. Impact frequency was the total number of impacts sustained in a given year. Impact acceleration was the average acceleration sustained in a given year. This analysis used one observation per year played for each participant.

### Multivariate, impact-level analyses

We used separate linear mixed-effect models to assess impact-level associations of impact acceleration in g’s (dependent variable) with impact characteristic, demographic, behavioral, and cognitive measures (independent variables), adjusted for total number of plays joined during the corresponding year. The dataset used contained one observation per impact occurrence. Because all impact characteristics were categorical, appropriate reference groups were selected.

### Hypothesis testing

To test our *a priori* hypotheses, separate models used: 1) impact frequency per year as the dependent variable, total ADHD symptom, inattention, and hyperactivity-impulsivity scores, and CPT measures as the independent variables, and the average number of plays joined each year as the covariate, 2) impact severity as the dependent variable, helmet-to-ground (relative to helmet-to-helmet) impacts as the independent variable, and total number of plays during the corresponding year as the covariate, and 3) impact severity as the dependent variable, special teams positions as the independent variable (relative to quarterback), and total number of plays during the corresponding year as the covariate. Other analyses were hypothesis-generating, adjusting for average number of plays each year in player-level models and total number of plays during the corresponding year in the remaining models. Because most analyses were hypothesis-generating, we did not correct p-values for multiple comparisons.

## Results

We recruited 53 participants at baseline (Table 1). We used player-level ANOVA and Pearson χ^2^ models to test differences in demographic, behavioral, and cognitive variables among players who discontinued football at any point during the 3 years (n = 15) and players who played in all 3 years (n = 38). We found no significant differences in age (p = 0.69), annual household income (p = 0.82), BMI (p = 0.47), push-up test time to recovery (p = 0.30), ethnicity (p = 0.42), race (p = 0.46), WASI IQ scores (p = 0.86), inattention symptom scores (p = 0.52), hyperactivity-impulsivity symptom scores (p = 0.88), total ADHD symptom scores (p = 0.74), CPT reaction time T-scores (p = 0.85), CPT omissions T-scores (p = 0.78), or CPT commissions T-scores (p = 0.46).

We used player-level analysis of variance to assess differences in demographic, behavioral, cognitive, and impact characterisitcs among the three cohorts. Cohorts did not differ by age (p = 0.73), household annual income (p = 0.99), BMI (p = 0.94), ethnicity (p = 0.61), or race (p = 0.87), nor did they differ significantly on inattention symptom scores (p = 0.67), hyperactivity-impulsivity symptom scores (p = 0.82), total ADHD symptom scores (p = 0.74), WASI IQ scores (p = 0.43), CPT reaction time T-scores (p = 0.51), or CPT omissions T-scores (p = 0.75). Cohort 3 had a significantly greater mean push-up time-to-recovery compared with Cohort 1 (mean difference = 0.83, p = 0.004) and Cohort 2 (mean difference = 1.12, p<0.001). Cohort 3 also had significantly higher mean CPT commission T-scores (mean difference = 7.69, p = 0.01) and detectability T-scores (mean difference = 8.0, p = 0.05) than Cohort 2. Cohorts did not differ by the frequency of impacts per year of play (p = 0.21), peak impact linear acceleration per year of play (p = 0.34), average number of plays joined each year of play (p = 0.33), or average number of games played per year of play (p = 0.19).

Across all 3 cohorts and years, 10,480 impacts were registered, 5,411 (52%) of which were false (Table 2, S1 Table), and 391 (4%) of which were deemed valid but collected from the Insite System, leaving 4,678 (45%) valid, in-game impacts from the HITS system. Most impacts were low to moderate in head acceleration (94% of all impacts; average impact acceleration was 29.3 g’s), were at the front of the head (54%), were a consequence of helmet-to-helmet contact (50%), and occurred during run plays (69%).

**Table 2:** Head impact characteristics accrued over 3 years of play for 3 combined cohorts.

**Player-level impact characteristics (N = 53 players)**	
Number of true impacts	4,678
Number of games	
Freshman	23
JV	33
Varsity	48
	**Mean (SD)**
Frequency of impacts per player (per year of play)	49.7 (66.4)
Peak linear acceleration (g/impact/year of play)	26.7 (7.8)
Number plays participated in per player (per year of play)	282.2 (248.5)
Number of total games played in per player (per year of play)	6.0 (2.3)
Impact rate (#/play)	0.2 (0.1)
**Impact-level characteristics (N = 4,678)**	**Mean (SD)**
Peak linear acceleration (g’s)	29.3 (17.8)
	**N (%)**
Impact acceleration bin	
Low (15–28.9 g)	2,959 (63)
Medium (29–62.9 g)	1,454 (31)
High (63+ g)	265 (6)
Skill level (# of impacts)	
Freshman	1,487 (32)
JV	1,473 (31)
Varsity	1,718 (37)
Location (# of impacts)	
Top	587 (12)
Front	2,514 (54)
Back	702 (15)
Side	875 (19)
External source (# of impacts)	
Helmet	2,303 (50)
Shoulder	719 (15)
Torso	705 (15)
Ground	482 (10)
Hand, Elbow	270 (6)
Knee, Leg, Foot	57 (1)
Ball	3 (0)
Whiplash/head acceleration	13 (0)
Unclear	126 (3)
Impact sequence (# of impacts)	
Primary	4,082 (87)
Secondary	489 (11)
Tertiary	95 (2)
Quaternary	12 (0)
Team (# of impacts)	
Offense	1,848 (40)
Defense	2,281 (49)
Special teams	549 (11)
Play type (# of impacts)	
Pass	889 (19)
Run	3,243 (69)
Special teams	544 (12)
Unclear	2 (0)

Six players across all 3 cohorts and years sustained 7 clinically-diagnosed concussions, 2 of which were diagnosed after an alert-triggering impact (i.e., an impact with an acceleration greater than 63 g’s) and therefore were identifiable on the video recordings. One concussion occurred when the player was playing defensive back during a pass play, occurred to the top of the head, was helmet-to-ground, and had an acceleration of 101.3 g’s. The second occurred when the player was playing on special teams, was the consequence of an impact to the front of the head, was helmet-to-helmet, and had an acceleration of 77.4 g’s. The other 5 concussions were diagnosed after a player removed himself from the game and reported symptoms to the athletic trainer. They therefore were not identifiable on video recordings.

### Risk for head impacts by player characteristics

We used negative binomial regression models to assess player-level associations of baseline demographic, behavioral, and cognitive characteristics with impact frequency, as over-dispersion was indicated in the Pearson’s Goodness of Fit statistic in the Poisson model (p<0.05) and in the significance test for the over-dispersion parameter (p’s<0.001). Player age and annual household income were not associated with impact frequency (p’s>0.32), likely because of the narrow range of these variables in our sample. Number of impacts per year associated positively with parent-reported severity of hyperactive-impulsive symptoms of ADHD [exp(β) = number of impacts per year 1.05 times (5%) higher per year per unit of symptom severity, 95% CI 1.04, 1.06, p = 0.01, Table 3] and objective measures of inattentiveness on the CPT, including detectability [exp(β) = number of impacts per year 1.02 times (2%) higher per year per T-score unit, 95% CI 1.01, 1.04, p = 0.02, Table 3] and omissions [exp(β) = number of impacts per year 1.003 times (0.003%) higher per year per T-score unit, 95% CI 1.001, 1.01, p = 0.01, Table 3].

**Table 3:** Associations of personal traits at baseline with head impact frequency accumulated across all years of play (N = 52 players).

	Head Impact Frequency (frequency of impacts/year)^a^	
	Exp(B) [95% CI]	p-value
*A priori analyses*		
ADHD symptom score	1.02 (0.99, 1.04)	0.08
Inattention symptom score	1.03 (0.99, 1.06)	0.13
Hyperactivity-impulsivity symptom score	**1.05 (1.04, 1.06)**	**0.01**
Connors’ CPT		
Detectability (T-score)	**1.02 (1.01, 1.04)**	**0.02**
Reaction time (T-score)	1.00 (0.98, 1.02)	0.93
Omissions (T-score)	**1.003 (1.001, 1.01)**	**0.01**
Commissions (T-score)	1.01 (0.98, 1.03)	0.35
*Hypothesis-generating analyses*		
BMI (kg/m^2^)^c^	1.01 (0.97, 1.04)	0.65
Push-up test recovery time (minutes)^b^	1.02 (0.98, 0.1.03)	0.90
IQ score	0.99 (0.97, 1.01)	0.44

**Note**: All models are adjusted for average number of plays joined per year. Beta values and the corresponding 95% CI were exponentiated to provide a more meaningful interpretation.

^a^ Negative binomial regression models used due to evidence of Poisson overdispersion.

^b^ N = 49

^c^ N = 51

Abbreviations: ADHD: Attention-Deficit/Hyperactivity Disorder, CPT: Continuous Performance Test Version 3.0, BMI: body mass index, IQ: Intelligence Quotient.

### Risk for head impacts by experience level

In multivariate, year-level mixed-effects models (data not shown in tables), compared to a player’s first year of play, the frequency of impacts was higher in the second (β = 33.4 more impacts, 95% CI 5.6, 61.1, p = 0.02) and third year of play (β = 50.9 more impacts, 95% CI = 11.6, 90.4, p = 0.01), adjusting for the total number of plays joined in each year. Year of play was not associated impact acceleration (p’s>0.17). Age was not associated with impact frequency or acceleration (p’s>0.71).

### Risk for head impacts by player position

Descriptive analyses revealed that higher rates of impacts per play were sustained in running backs (46% of plays), centers (28% of plays), kick-punt returners (25% of plays), linebackers (24% of plays), defensive linemen (23% of plays), and other special team players (18% of plays) compared with other positions (S2 Table).

**Fig 3:**
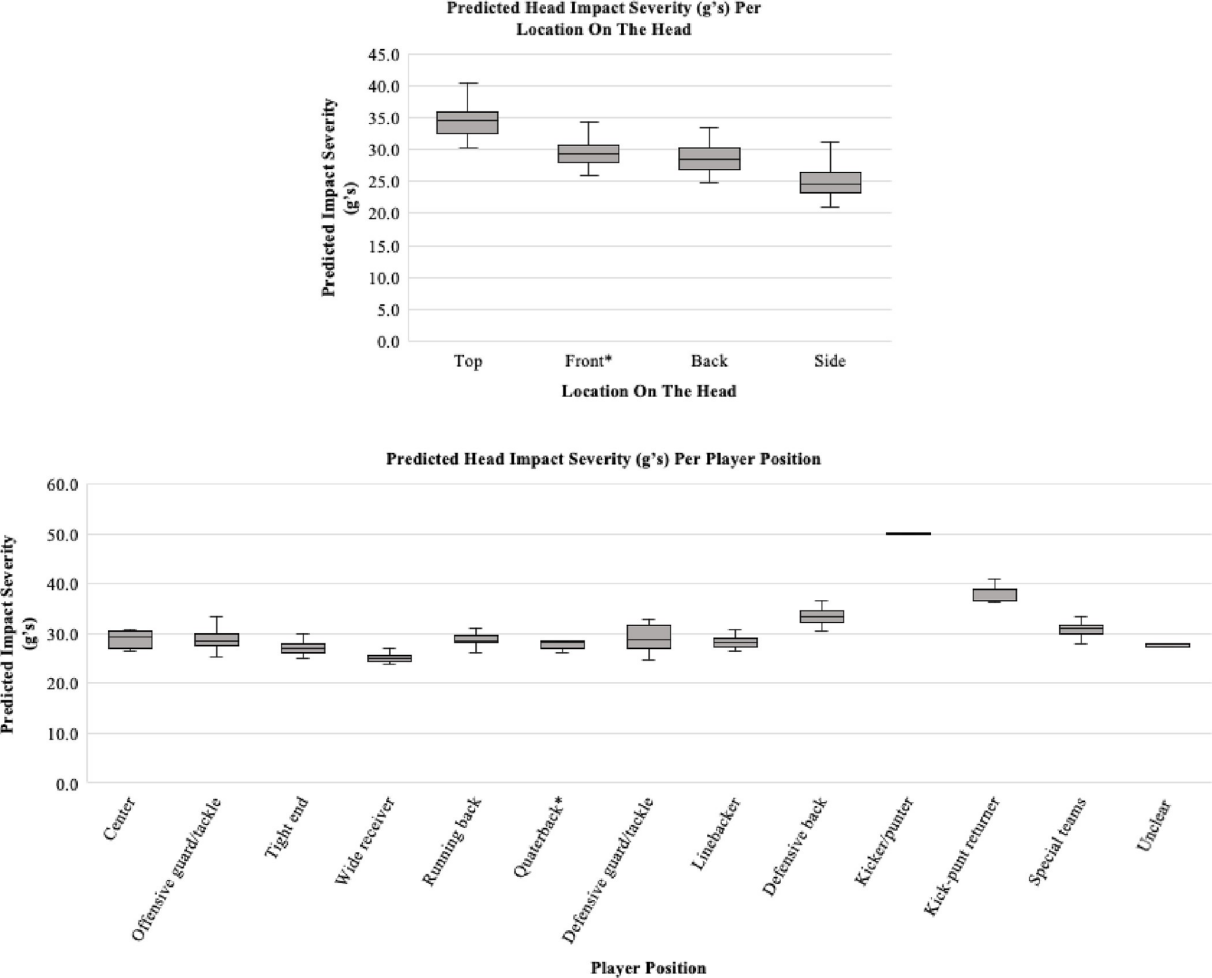
Boxplots of the predicted impact severity per location on the head using acceleration in g’s (top) and player position using acceleration in g’s (bottom). All datapoints are adjusted for total number of plays participated in for the corresponding year. * = reference category used in the mixed-effect model.

Table 4 presents multivariate, impact-level associations between impact characteristics and acceleration in g’s. In multivariate, impact-level mixed-effects models of player position with head acceleration, we used impacts sustained by the quarterback as a reference because special rules aim to protect this player from excessive hits. Kickers/punters sustained higher acceleration impacts than the quarterback (β = 21.5 g’s higher, 95% CI 7.6, 35.4, p = 0.002, Table 4) and higher acceleration impacts than other positions on special teams when excluding the kick-punt returner (β = 18.4 g’s higher, 95% CI 5.1, 31.7, p = 0.007, Table 4 notes). Compared with quarterbacks, head acceleration was also greater for impacts sustained by defensive backs (β = 4.9 g’s higher, 95% CI -0.04, 9.9, p = 0.05, Table 4) and kick-punt returners (β = 9.3 g’s higher, 95% CI 0.8, 17.9, p = 0.03, Table 4) (Fig 3). Acceleration was greater for impacts occurring on special teams compared to offense (β for offense = -2.2 g’s less compared to special teams, 95% CI -4.0, -0.4, p = 0.02, Table 4) and defense (β for defense = -1.9 g’s less compared to special teams, 95% CI -3.6, -0.2, p = 0.03, Table 4). Impacts during special teams plays were greater in acceleration than run plays (β for run plays = -2.0 g’s less compared to special teams plays, 95% CI -3.7, -0.3, p = 0.02, Table 4).

**Table 4:** Impact-level associations of impact characteristics with impact acceleration accrued over 3 years of play for 3 combined cohorts.

	Impact Acceleration (g’s) N = 4,678 impacts		
	β (95% CI)	p-value	Cohen’s d
Skill level			
Freshman	--	--	--
JV	**2.5 (0.5, 4.4)**	**0.01**	**0.1**
Varsity	**2.9 (0.6, 5.2)**	**0.01**	**0.2**
Location			
Top	**4.4 (2.8, 6.0)**	**<0.001**	**0.3**
Front	--	--	--
Back	-1.1 (-2.6, 0.4)	0.14	-0.1
Side	**-4.9 (-6.3, -3.6)**	**<0.001**	**-0.3**
External source			
Helmet	--	--	--
Shoulder	-1.2 (-2.7, 0.3)	0.13	-0.1
Torso	0.2 (-1.3, 1.8)	0.77	0.01
Ground	1.7 (-0.4, 3.5)	0.06	0.1
Hand and elbow	-2.1 (-4.3, 0.2)	0.07	-0.1
Knee, Leg, Foot	2.5 (-2.2, 7.2)	0.29	0.1
Ball	2.2 (-17.8, 22.2)	0.83	0.1
Whiplash/head acceleration	**-12.0 (-21.7, -2.4)**	**0.01**	**-0.7**
Unclear	**-6.7 (-9.9, -3.5)**	**<0.001**	**-0.4**
Impact sequence			
Primary	--	--	--
Secondary	**-1.8 (-3.5, -0.1)**	**0.03**	**-0.1**
Tertiary	**-3.7 (-7.3, -0.04)**	**0.05**	**-0.2**
Quaternary	**-11.1 (-21.2, -1.1)**	**0.03**	**-0.6**
Unclear	N/A	N/A	N/A
Position			
Center	1.7 (-3.5, 7.0)	0.52	0.1
Offensive guard/tackle	1.1 (-3.9 6.1)	0.67	0.1
Tight end	0.6 (-4.6, 5.8)	0.82	0.03
Wide receiver	-2.7 (-9.1, 3.8)	0.42	-0.2
Running back	1.2 (-3.7, 6.2)	0.62	0.1
Quarterback	--	--	--
Defensive tackle/end	0.5 (-4.4, 5.4)	0.84	0.03
Linebacker	0.9 (-3.8, 5.7)	0.71	0.1
Defensive back	**4.9 (-0.04, 9.9)**	**0.05**	**0.3**
Kicker/punter	**21.5 (7.6, 35.4)** ^**a**^	**0.002**	**1.2**
Kick-punt returner	**9.3 (0.8, 17.9)**	**0.03**	**0.5**
Special teams	3.0 (-1.8, 7.9)	0.22	0.2
Unclear	N/A	N/A	N/A
Team			
Offense	**-2.2 (-4.0, -0.4)** ^**b**^	**0.02**	**-0.1**
Defense	**-1.9 (-3.6, -0.2)**	**0.03**	**-0.1**
Special teams	--	--	--
Unclear	N/A	N/A	N/A
Play type			
Pass	-1.9 (-3.8, 0.1)	0.06	-0.1
Run	**-2.0 (-3.7, -0.3)** ^**c**^	**0.02**	**-0.1**
Special teams	--	--	--
Unclear	-10.5 (-35.2, 14.2)	0.41	0.6

**Note**: All models were adjusted for total number of plays joined for the corresponding year;—indicates the reference group; N/A: not applicable due to the ability to classify all impact characteristics within the model.

**Unclear**: some impact characteristics (i.e., external source, impact sequence, position, team, play type) were unable to be derived because they were unviewable (e.g., a heap of players); unviewable characteristics were recorded as unclear.

^a^In post-hoc analyses, kickers/punters sustained higher acceleration impacts compared to special teams positions using acceleration (β = 18.41 g’s higher, 95% CI 5.09, 31.72, p = 0.007).

^b^In post-hoc analyses, playing defense was not associated with impact acceleration in g’s compared to playing offensive (β for defense = 0.29, 95% CI -0.94, 1.52, p = 0.64).

^c^In post-hoc analyses, run plays were not significantly associated with impact acceleration in g’s compared to pass plays (β for run plays = -0.12, 95% CI -1.45, 1.21, p = 0.86)

### Risk for head impacts by level of play

Multivariate, impact-level mixed-effects models were used to assess associations of level of play with head acceleration. Compared to participants on the freshman team, those on JV sustained higher acceleration impacts (β = 2.5 g’s higher, 95% CI 0.6, 4.4, p = 0.01, Table 4), as did players on varsity (β = 2.9 g’s higher, 95% CI 0.6, 5.2, p = 0.01, Table 4).

### Risk for head impacts by type of contact

In multivariate, impact-level mixed-effects models of type and location of contact with head impact acceleration, we selected front-of-the-head and helmet-to-helmet impacts as the reference groups since front-of-the-head impacts have been more often associated with concussions [50, 51] and helmet-to-helmet impacts have been associated with high-acceleration impacts [52]. Impacts were greater in acceleration to the top of the head than to the front (β = 4.4 g’s higher, 95% CI 2.8, 6.0, p<0.001) (Table 4 and Fig 3).

Multivariate, impact-level mixed-effects models were used to assess associations of impact sequence with impact acceleration. Compared to primary impacts, secondary (β = -1.8 g’s less, 95% CI -3.5, -0.1, p = 0.03, Table 4), tertiary (β = -3.7 g’s less, 95% CI -7.3, -0.04, p = 0.05, Table 4), and quaternary impacts (β = -11.1 g’s less, 95% CI -21.2, -1.1, p = 0.03, Table 4) were lower in acceleration. In multivariate, impact-level mixed-effects models, associations of demographic, behavioral, and cognitive measures with impact acceleration were null (p’s>0.24).

## Discussion

As hypothesized, players with more severe parent-reported ADHD symptoms before starting football subsequently sustained more head impacts. Experience and level of play were associated with more frequent impacts and with more forceful, respectively. Head impacts while playing defensive back, kick-punt returner, and kicker/punter were of greater force than impacts for comparable players at other positions. Impacts to the top of the head and the ground generated impacts with higher acceleration.

The number of true compared with false impacts was lower than in prior studies [26, 53, 54], which video recordings showed were attributable to players often tossing their helmets on the ground or hitting them when not on the field during a play. Acceleration values were similar to those reported previously in male high school [26, 27, 50, 54–56] and college football players [56, 57]. Our sample sustained more impacts in later years of play than in prior studies [27, 28, 53, 54, 58]. Most prior studies used the same HITS sensors [27, 28, 53, 54], so the larger number of impacts per year in our study is unlikely to derive from differences in measurement technology. Many participants played multiple positions and therefore joined more plays, which could count for our greater frequency of head impacts. We are unable to compare impact rate per play to other studies because prior studies did not record the numbers of plays per player [26, 50, 55].

Parent reports of player ADHD symptoms and objective measures of inattention were associated with sustaining more impacts per year of play. Adolescents who enter football with symptoms of ADHD may be at higher risk for sustaining impacts in the future. ADHD symptoms have been associated previously with concussive events in male college and youth (9–18 years) football players [59, 60] and in male and female college athletes of various sports [61]. Those with more severe ADHD symptoms may be less attentive to tackling instructions, or more impulsive and therefore more likely to engage in head collisions without forethought [62]. Players with ADHD may also have difficulties with motor control, balance, and coordination [63], which could impair their ability to avoid tackles or to tackle with proper form. Coaches and staff who review an athlete’s pre-participation exam should consider providing specialized instruction to athletes who have a history of ADHD, such as periodic checks for proper tackling, or structuring practices to enhance their engagement and hone tackling skills [64].

Players sustained more frequent head impacts with each subsequent year of play. This finding stands in contrast to one prior study that reported a trend toward a declining number of impacts with each year of play in male high school football players [65]. Level of play was a better predictor of impact acceleration, as JV and varsity players sustained more forceful impacts than the freshman players, though acceleration did not differ significantly between JV and varsity players. In contrast to prior studies [30, 31, 59, 66], age did not associate with impact frequency or acceleration, perhaps because of the limited variance in our age variable. Moreover, because players progress each year in age, physical size, skill, and their confidence in tackling, these variables are difficult to disentangle statistically, and any one of these characteristics could contribute to more frequent and more forceful impacts. Future studies should consider investigating the role of confidence and self-efficacy on head impacts, as these may mediate the associations of experience and skill with head impacts.

Defensive backs and kick-punt returners sustained more forceful impacts compared to quarterbacks, likely because these players typically accelerate before contact. Kickers/punters compared to quarterbacks and special teams positions, and special teams players compared to those on defense or offense, also sustained higher force impacts. Consistent with our findings, a prior study in youth (10–13 years) reported that special teams plays yielded higher acceleration impacts compared to running and pass plays [29]. Another study of adolescents, however, suggested that running backs were at the highest risk for concussion [67]. Kickers/punters are generally not viewed as high-risk for head impacts, as tackling the kicker/punter is heavily penalized. However, our detailed video analyses confirmed that the recorded impacts to kickers/punters were true, in-game impacts, usually sustained when the kicker or punter was attempting to pursue or tackle the opposing kick returner. Although our sample size was small, our findings highlight the need to monitor all players on the field, regardless of player position. Kickers/punters may be on the field for only 3–5 plays per game, but they are at risk for forceful impacts. Policymakers may want to consider changes to the current kick-off rules in high school to reduce head impact burdens for these players. For example, the National Football League recently implemented a rule stating that if a fair catch is completed behind the 25-yard line, players can have the first down start at the 25-yard line [68]. This rule minimizes concussion risk because it prevents players from running full speed at each other across the field. High school football policymakers could consider integrating this rule to enhance player safety. High school football policymakers could also consider eliminating kickoffs, given the greater vulnerability of the adolescent brain.

Top-of-the-helmet impacts were more forceful compared to the front of the head. Consistent with our findings, prior studies have found that although impacts most frequently occur to the front of the head, top-of-the-head impacts are most forceful in male high school [28, 54] and youth [66] football players. Video analyses showed that most (53%) impacts to the top-of-the-head occurred when blocking. In exploratory analyses, centers and offensive guards/tackles—positions in which the primary objective is to block—were more likely than wide receivers, tight ends, or linebackers to sustain impacts to the top of the head (data not shown). Helmet-to-torso collisions and run plays were also more likely to produce top-of-the-head impacts (data not shown). Our video analyses suggest that most of these impacts are sustained when offensive linemen drop their heads down and then lead with the top of their head during a block, rather than keeping their head up as suggested for proper form. Video analyses also suggest that players will less often extend upward from a 3-point stance while lowering their head during a block, which delivers more force to the top of their head. Further instruction in proper blocking techniques could reduce these impacts in offensive linemen. In addition, 35% of impacts to the top of the head occurred while tackling, suggesting that improper tackling techniques contribute to these impacts. Because forceful impacts to the top of the head are known to increase the risk concussion and cervical spine injury [51], coaches should emphasize techniques that keep the head up during blocking and tackling. Engineering helmets to better cushion the top of the head could also attenuate head acceleration.

Strengths of this study include its longitudinal design, use of in-helmet sensors to obtain head impact acceleration, and multi-camera video recordings followed by careful and detailed video analyses to characterize individual impacts. We used objective measures for many of our variables, whereas prior studies have largely relied on self-reports [59–61]. However, the generalization of our results may be limited due to our modest sample size, limited numbers of participants at some player positions, and players identified in a single high school football program. We also recruited youth who had no prior history of concussion and no history of participation in contact sports to improve the internal validity of our findings. Nevertheless, excluding youth who previously played contact sports may also limit the generalizability of our findings, as youth often play contact sports prior to high school. We estimate that 5 youth were excluded for this reason, and deem its influence on generalizability of our findings was minor. We also had a high rate of missed follow-up assessments due to the COVID-19 pandemic, which limited our ability to conduct repeated measures analyses and contributed to the limited generalizability of our findings.

High school football players sustain head impacts at a high frequency and over a wide range of force. Our findings can be used by coaches, officials, trainers, equipment engineers, and policymakers to guide evidence-based modification of screening procedures, protective gear, training, technique, game structure, and rules to reduce neurocognitive impairments associated with head impacts.

## Supporting information

S1 File Methods, results, and discussion on statistical models that combined insite and HITS head impact data. (DOCX)

S1 Table Head impact characteristics within each cohort per year of play using impacts derived from the HITS data. Unclear: some impact characteristics (i.e., external source, play type) were unable to be derived because they were unviewable (e.g., a heap of players); unviewable characteristics were recorded as unclear. ^a^ N = 17 players sustained impacts; n = 3 played and did not sustain any impacts. ^b^ N = 14 players sustained impacts. ^c^ N = 10 players sustained impacts; n = 1 played and did not sustain any impacts. ^d^ N = 19 players sustained impacts; n = 1 played and did not sustain any impacts. ^e^ N = 11 players sustained impacts; n = 2 players were on the team but did not play; n = 1 played and did not sustain any impacts. ^f^ N = 12 players sustained impacts; n = 1 player was on the team but did not play.
(DOCX)

S2 Table Impact characteristics by position using impacts derived from the HITS data. (DOCX)
