# Supplementary material for: Longitudinal, prospective study of head impacts in male high school football players
Source: PLoS One. 2023 Sep 8;18(9):e0291374. doi: 10.1371/journal.pone.0291374 (PMC10490840; doi:10.1371/journal.pone.0291374)
Supplement: S1 File — (DOCX) [file pone.0291374.s001.docx]

**Supplement 1. Methods, Results, and Discussion on Statistical Models That Combined Insite and HITS Head Impact Data**

**Methods**

**Head impacts**

Because the Insite System grouped the acceleration of head impacts into 3 bins (i.e., low, medium, and high), we grouped the HITS acceleration and location data for each impact (originally recorded in g’s) into the same bins. These groupings thereby harmonized the InSite and HITS data precisely and allowed us to use all available data from both measurement systems. The two sensor systems had identical thresholds of 15g for recording an impact.

**Statistical analyses**

We used the same statistical methods presented in the manuscript for the analyses using head impacts from both the Insite System and the HITS. However, since our outcome was ordinal for the multivariate, impact-level analyses, we used separate mixed-effect ordinal logistic regression models to assess impact-level associations of impact acceleration bin (ordinal dependent variable) with impact characteristic, demographic, behavioral, and cognitive measures (independent variables), adjusted for total number of plays during the corresponding year.

**Results**

After combining head impacts sustained with the Insite System and the HITS, a total of 5,069 (48%) valid, in-game impacts were sustained among the 53 players. Similar to the HITS-only data, most impacts were low to moderate in head acceleration (95% of all impacts), were at the front of the head (55%), were a consequence of helmet-to-helmet contact (50%), and occurred during run plays (70%) (S1 Table 1).

**S1 Table 1. Head Impact Characteristics Accrued Over 3 Years of Play for 3 Combined Cohorts**

| **Player-level impact characteristics (N=53 players)** |  |
| --- | --- |
| Number of true impacts | 5,069 |
| Number of games |  |
| Freshman | 27 |
| JV | 33 |
| Varsity | 48 |
|  | **Mean (SD)** |
| Frequency of impacts per player (per year of play) | 45.7 (58.7) |
| Number plays participated in per player (per year of play) | 289.2 (241.0) |
| Number of total games played in per player (per year of play) | 6.9 (2.4) |
| Impact rate (#/play) | 0.1 (0.1) |
| **Impact-level characteristics (N=5,069)** | **N (%)** |
| Impact acceleration bin |  |
| Low (15-28.9 g) | 3,141 (62) |
| Medium (29-62.9 g) | 1,655 (33) |
| High (63+ g) | 273 (5) |
| Skill level (# of impacts) |  |
| Freshman | 1, 878 (37) |
| JV | 1, 473 (29) |
| Varsity | 1, 718 (34) |
| Location (# of impacts) |  |
| Top | 606 (12) |
| Front | 2, 787 (55) |
| Back | 714 (14) |
| Side | 962 (19) |
| External source (# of impacts) |  |
| Helmet | 2, 546 (50) |
| Shoulder | 790 (16) |
| Torso | 735 (15) |
| Ground | 509 (10) |
| Hand, Elbow | 277 (6) |
| Knee, Leg, Foot | 63 (1) |
| Ball | 3 (0) |
| Whiplash/head acceleration | 13 (0) |
| Unclear | 133 (2) |
| Impact sequence (# of impacts) |  |
| Primary | 4, 452 (88) |
| Secondary | 505 (10) |
| Tertiary | 97 (2) |
| Quaternary | 12 (0) |
| Unclear | 3 (0) |
| Team (# of impacts) |  |
| Offense | 2, 054 (41) |
| Defense | 2, 433 (48) |
| Special teams | 579 (11) |
| Unclear | 3 (0) |
| Play type (# of impacts) |  |
| Pass | 954 (19) |
| Run | 3, 528 (70) |
| Special teams | 573 (11) |
| Unclear | 14 (0) |

**Risk for head impacts by player characteristics**

We used negative binomial regression models to assess player-level associations of baseline demographic, behavioral, and cognitive characteristics with impact frequency, as over-dispersion was indicated in the Pearson’s Goodness of Fit statistic in the Poisson model (p<0.05) and in the significance test for the over-dispersion parameter (p’s<0.001). Player age and annual household income were not associated with impact frequency (p’s>0.74), likely because of the narrow range of these variables in our sample. Number of impacts per year associated positively with parent-reported severity of hyperactive-impulsive symptoms of ADHD [exp(β)=number of impacts per year 1.05 times (5%) higher per year per unit of symptom severity, 95% CI 1.01, 1.09, p=0.03, S1 Table 2] and objective measures of inattentiveness on the CPT, including detectability [exp(β)= number of impacts per year 1.02 times (2%) higher per year per T-score unit, 95% CI 1.002, 1.04, p=0.03, Table S1 Table 2] and omissions [exp(β)= number of impacts per year 1.02 times (2%) higher per year per T-score unit, 95% CI 1.01, 1.03, p=0.01, S1 Table 2].

**S1 Table 2. Associations of Personal Traits at Baseline with Head Impact Frequency Accumulated Across All Years of Play (N=52 players)**

|  | **Head Impact Frequency**  **(frequency of impacts/year)^a^** | |
| --- | --- | --- |
|  | **Exp(B) [95% CI]** | **p-value** |
| *A priori analyses* |  |  |
| ADHD symptom score | 1.02 (0.99, 1.04) | 0.06 |
| Inattention symptom score | 1.02 (0.99, 1.06) | 0.17 |
| Hyperactivity-impulsivity symptom score | **1.05 (1.01, 1.09)** | **0.03** |
| Connors’ CPT |  |  |
| Detectability (T-score) | **1.02 (1.002, 1.04)** | **0.03** |
| Reaction time (T-score) | 1.003 (0.98, 1.03) | 0.78 |
| Omissions (T-score) | **1.02 (1.01, 1.03)** | **0.01** |
| Commissions (T-score) | 1.01 (0.98, 1.04) | 0.39 |
| *Hypothesis-generating analyses* |  |  |
| BMI (kg/m^2^)^c^ | 1.01 (0.97, 1.04) | 0.74 |
| Push-up test recovery time (minutes)^b^ | 1.23 (0.95, 1.58) | 0.12 |
| IQ score | 0.99 (0.97, 1.01) | 0.18 |

**Note**: All models are adjusted for average number of plays per year participated in. Beta values and the corresponding 95% CI were exponentiated to provide a more meaningful interpretation.

^a^ Negative binomial regression models used due to evidence of Poisson overdispersion.

^b^ N=49

^c^ N=51

**Risk for head impacts by experience level**

In multivariate, year-level mixed-effects models (data not shown in tables), compared to a player’s first year of play, the frequency of impacts was higher in the second (β=18.2 more impacts, 95% CI 1.03, 35.4, p=0.05) and third year of play (β=42.2 more impacts, 95% CI=17.7, 66.6, p=0.002), adjusting for the total number of plays in each year. Year of play was not associated with the acceleration bin (p’s>0.14). Age was not associated with impact frequency or acceleration (p’s>0.30).

**Risk for head impacts by player position**

S1 Table 3 presents multivariate, impact-level associations between impact characteristics and acceleration measured on an ordinal scale. Player position was not associated with impact acceleration (p’s>0.13, S1 Table 3). Play type (p’s>0.14, Table S1 Table 3) and team (p’s>0.19, S1 Table 3) were not associated with impact acceleration.

**S1 Table 3. Impact-Level Associations of Impact Characteristics with Impact Acceleration Accrued Over 3 Years of Play for 3 Combined Cohorts**

|  | **Ordinal Outcome Models**  **(All impacts)** | | | |
| --- | --- | --- | --- | --- |
|  | **Impact Acceleration Bin**  **(1=low, 2=medium, 3=high)**  **N=5,069 impacts** | | | |
|  | **β (95% CI)** | **p-value** | **OR** | **Cohen’s d** |
| Skill level |  |  |  |  |
| Freshman | -- | -- | -- | -- |
| JV | **0.3 (0.01, 0.5)** | **0.05** | **1.3** | **0.1** |
| Varsity | 0.1 (-0.2, 0.4) | 0.57 | 1.1 | 0.1 |
| Location |  |  |  |  |
| Top | **0.4 (0.2, 0.6)** | **<0.001** | **1.5** | **0.2** |
| Front | -- | -- | -- | -- |
| Back | **-0.2 (-0.4, 0.1)** | **0.007** | **0.8** | **-0.1** |
| Side | **-0.7 (-0.8, 0.5)** | **<0.001** | **0.5** | **-0.4** |
| External source |  |  |  |  |
| Helmet | -- | -- | -- | -- |
| Shoulder | -0.2 (-0.3, 0.01) | 0.06 | 0.9 | -0.1 |
| Torso | 0.04 (-0.1, 0.2) | 0.65 | 1.0 | 0.02 |
| Ground | **0.2 (0.02, 0.4)** | **0.03** | **1.2** | **0.1** |
| Hand and elbow | -0.1 (-0.4, 0.1) | 0.38 | 0.9 | -0.1 |
| Knee, Leg, Foot | **0.5 (0.1, 1.0)** | **0.03** | **1.7** | **0.3** |
| Ball | 0.8 (-1.3, 2.9) | 0.46 | 2.2 | 0.4 |
| Whiplash/head acceleration | -1.9 (-4.0, 0.2) | 0.07 | 0.2 | -1.0 |
| Unclear | -0.4 (-0.7, 0.03) | 0.07 | 0.7 | -0.2 |
| Impact sequence |  |  |  |  |
| Primary | -- | -- | -- | -- |
| Secondary | **-0.4 (-0.6, -0.2)** | **0.001** | **0.7** | **-0.2** |
| Tertiary | -0.3 (-0.8, 0.1) | 0.15 | 0.7 | -0.2 |
| Quaternary | -1.2 (-2.7, 0.3) | 0.12 | 0.3 | -0.7 |
| Unclear | -0.4 (-2.8, 2.1) | 0.78 | 0.9 | -0.1 |
| Position |  |  |  |  |
| Center | -0.2 (-0.7, 0.4) | 0.60 | 0.9 | -0.1 |
| Offensive guard/tackle | 0.1 (-04, 0.7) | 0.62 | 1.2 | 0.1 |
| Tight end | -0.3 (-0.9, 0.3) | 0.34 | 0.8 | -0.2 |
| Wide receiver | 0.01 (-0.7, 0.7) | 0.99 | 1.0 | 0.01 |
| Running back | 0.1 (-0.5, 0.6) | 0.84 | 1.1 | 0.03 |
| Quarterback | -- | -- | -- | -- |
| Defensive tackle/end | -0.1 (-0.6, 0.5) | 0.79 | 0.9 | -0.04 |
| Linebacker | -0.1 (-0.6, 0.4) | 0.67 | 0.9 | -0.1 |
| Defensive back | 0.1 (-0.4, 0.7) | 0.63 | 1.1 | 0.1 |
| Kicker/punter | 1.2 (-0.3, 2.7) | 0.13 | 3.2 | 0.6 |
| Kick-punt returner | -0.1 (-1.0, 0.8) | 0.82 | 0.9 | -0.1 |
| Special teams | 0.1 (-0.4, 0.6) | 0.74 | 1.1 | 0.1 |
| Unclear | -0.4 (-2.8, 2.1) | 0.78 | 0.7 | -0.2 |
| Team |  |  |  |  |
| Offense | -0.1 (-0.3, 0.1) | 0.51 | 0.9 | -0.04 |
| Defense | -0.1 (-0.3, 0.1) | 0.19 | 0.9 | -0.1 |
| Special teams | -- | -- | -- | -- |
| Unclear | -0.4 (-2.9, 2.0) | 0.74 | 0.7 | -0.2 |
| Play type |  |  |  |  |
| Pass | -0.2 (-0.4, 0.1) | 0.14 | 0.9 | -0.1 |
| Run | -0.1 (-0.3, 0.1) | 0.57 | 1.0 | -0.03 |
| Special teams | -- | -- | -- | -- |
| Unclear | -0.1 (-1.2, 0.9) | 0.82 | 0.9 | 0.9 |

Note: All models were adjusted for total number of plays participated in for the corresponding year; -- indicates the reference group; N/A: not applicable due to the ability to classify all impact characteristics within the model.

Unclear: some impact characteristics (i.e., external source, impact sequence, position, team, play type) were unable to be derived because they were unviewable (e.g., a heap of players); unviewable characteristics were recorded as unclear.

**Risk for head impacts by level of play**

Multivariate, impact-level mixed-effects models were used to assess associations of level of play with head acceleration. Compared to participants on the freshman team, those on JV sustained higher acceleration impacts using ordinal impact bin (β=0.3, 95% CI 0.01, 0.5, p=0.05, S1 Table 3).

**Risk for head impacts by type of contact**

Multivariate, impact-level mixed-effects models were used to assess associations of type of contact with head acceleration. Impacts were greater in acceleration to the top of the head than to the front using ordinal impact bin (β=0.4, 95% CI 0.2, 0.6, p<0.001, S1 Table 3). Impacts were also greater in acceleration for the head striking both the ground (β=0.2, 95% CI 0.02, 0.4, p=0.03, S1 Table 3) or the knee, leg, or foot of another player (β=0.5, 95% CI 0.1, 1.0, p=0.03, Table S1 Table 3) compared to helmet-to-helmet impacts using ordinal impact bin. Secondary impacts were lower in acceleration than primary impacts when using ordinal impact bin (β=-0.4, 95% CI -0.6, -0.2, p=0.001, S1 Table 3). In multivariate, impact-level mixed-effects models, associations of demographic, behavioral, and cognitive measures with impact bin were null (p’s>0.24).

**Discussion**

The only difference between models using the Insite System and HITS data compared to models only using the HITS data was that acceleration was greater for impacts from the ground or another player’s leg, knee, or foot than for helmet-to-helmet impacts. In the HITS-only models, this association was non-significant. Video analyses indicated that helmet-to-helmet impacts tended to be glancing blows, likely a consequence of the intense coaching focus on reducing direct, helmet-to-helmet hits. Tackling techniques should emphasize aiming the tackling shoulder to the torso to reduce the force of head impacts. Helmet-to-ground impacts may warrant a lower threshold for screening for concussion.
