# Supplementary material for: Longitudinal, prospective study of head impacts in male high school football players
Source: PLoS One. 2023 Sep 8;18(9):e0291374. doi: 10.1371/journal.pone.0291374 (PMC10490840; doi:10.1371/journal.pone.0291374)
Supplement: S1 Table — Unclear: some impact characteristics (i.e., external source, play type) were unable to be derived because they were unviewable (e.g., a heap of players); unviewable characteristics were recorded as unclear. a N = 17 players sustained impacts; n = 3 played and did not sustain any impacts. b N = 14 players sustained impacts. c N = 10 players sustained impacts; n = 1 played and did not sustain any impacts. d N = 19 players sustained impacts; n = 1 played and did not sustain any impacts. e N = 11 players sustained impacts; n = 2 players were on the team but did not play; n = 1 played and did not sustain any impacts. f N = 12 players sustained impacts; n = 1 player was on the team but did not play. (DOCX) [file pone.0291374.s002.docx]

**Supplement 2. Head Impact Characteristics Within Each Cohort Per Year of Play Using Impacts Derived From the HITS Data**

|  | **Cohort 1** | | | **Cohort 2** | | **Cohort 3** |
| --- | --- | --- | --- | --- | --- | --- |
| **Player-level impact characteristics (N=53)** | **Year 1**  **(n=20)** | **Year 2**  **(n=14)** | **Year 3**  **(n=11)** | **Year 1**  **(n=20)** | **Year 2**  **(n=14)** | **Year 1**  **(n=13)** |
| Total number of true impacts | 581 | 1,284 | 1,079 | 641 | 637 | 456 |
| Total number of games |  |  |  |  |  |  |
| Freshman | 4 | -- | -- | 9 | **--** | 8 |
| JV | -- | 9 | 12 | -- | 12 | -- |
| Varsity | -- | 10 | 14 | 10 | 14 | -- |
|  | **Mean (SD)** | **Mean (SD)** | **Mean (SD)** | **Mean (SD)** | **Mean (SD)^e^** | **Mean (SD)^f^** |
| Frequency of impacts per player | 32.8 (32.6) | 89.3 (105.3) | 97.9 (144.8) | 32.2 (36.1) | 51.9 (59.3) | 35.1 (38.4) |
| Peak linear acceleration (g/impact) | 26.7 (5.8) | 28.8 (5.2) | 26.6 (9.6) | 27.5 (6.8) | 27.1 (9.6) | 29.1 (6.6) |
| Number of total plays participated in | 147.7 (103.7) | 369.9 (307.6) | 339.2 (395.4) | 232.1 (217.4) | 316.5 (292.8) | 249.3 (236.9) |
| Number of total games played in | 3 (0.91) | 6.7 (2.6) | 9.5 (3.5) | 6.6 (2.5) | 5.9 (3.6) | 6.2 (2.5) |
| Impact rate (#/play) | 0.5 (0.3) | 0.2 (0.1) | 0.3 (0.2) | 0.1 (0.1) | 0.1 (0.1) | 0.2 (0.1) |
| **Impact-level characteristics (N=4,678)** | **Year 1**  **(n=581)^a^** | **Year 2**  **(n=1,284)^b^** | **Year 3**  **(n=1,079)^c^** | **Year 1**  **(n=641)^d^** | **Year 2**  **(n=637)^e^** | **Year 1**  **(n=456)^f^** |
|  | **Mean (SD)** | **Mean (SD)** | **Mean (SD)** | **Mean (SD)** | **Mean (SD)** | **Mean (SD)** |
| Peak linear acceleration (g’s) | 28.3 (17.9) | 28.9 (16.8) | 29.8 (18.2) | 28.9 (18.2) | 30.3 (19.5) | 29.1 |
|  | **N(%)** | **N(%)** | **N(%)** | **N(%)** | **N(%)** | **N(%)** |
| Impact acceleration bin |  |  |  |  |  |  |
| Low (15-28.9 g) | 393 (68) | 803 (63) | 666 (62) | 422 (66) | 396 (62) | 312 (68) |
| Medium (29-62.9 g) | 153 (26) | 423 (33) | 355 (33) | 174 (27) | 195 (31) | 90 (20) |
| High (63+ g) | 35 (6) | 58 (4) | 58 (5) | 45 (7) | 46 (7) | 54 (12) |
| Skill level (# of impacts) |  |  |  |  |  |  |
| Freshman | 581 (100) | -- | -- | 450 (70) | -- | 456 (100) |
| JV | -- | 791 (62) | 242 (22) | -- | 440 (69) | -- |
| Varsity | -- | 493 (38) | 837 (78) | 191 (30) | 197 (31) | -- |
| Location (# of impacts) |  |  |  |  |  |  |
| Top | 57 (10) | 119 (9) | 137 (13) | 71 (11) | 71 (11) | 132 (29) |
| Front | 344 (59) | 779 (61) | 552 (51) | 338 (53) | 313 (49) | 188 (41) |
| Back | 82 (14) | 178 (14) | 170 (16) | 129 (20) | 115 (18) | 28 (6) |
| Side | 98 (17) | 208 (16) | 220 (20) | 103 (16) | 138 (22) | 108 (24) |
| External source (# of impacts) |  |  |  |  |  |  |
| Helmet | 349 (60) | 694 (54) | 442 (40) | 370 (58) | 233 (37) | 215 (47) |
| Shoulder | 74 (13) | 177 (14) | 171 (16) | 97 (15) | 162 (25) | 38 (8) |
| Torso | 53 (9) | 157 (12) | 265 (25) | 44 (7) | 110 (17) | 76 (17) |
| Ground | 39 (7) | 130 (10) | 129 (12) | 47 (7) | 71 (12) | 66 (15) |
| Hand, Elbow | 34 (6) | 96 (8) | 40 (4) | 48 (8) | 24 (5) | 28 (6) |
| Knee, Leg, Foot | 13 (2) | 12 (1) | 7 (1) | 17 (3) | 4 (1) | 4 (1) |
| Ball | 0 (0) | 2 (0) | 0 (0) | 1 (0) | 0 (0) | 0 (0) |
| Whiplash/head acceleration | 0 (0) | 0 (0) | 0 (0) | 13 (2) | 0 (0) | 0 (0) |
| Unclear | 19 (3) | 16 (1) | 25 (2) | 4 (0) | 33 (5) | 29 (6) |
| Impact sequence (# of impacts) |  |  |  |  |  |  |
| Primary | 506 (87) | 1,104 (86) | 916 (85) | 563 (88) | 587 (92) | 406 (89) |
| Secondary | 63 (11) | 152 (12) | 123 (11) | 68 (11) | 40 (6) | 43 (9) |
| Tertiary | 12 (2) | 23 (2) | 36 (3) | 10 (1) | 8 (1) | 6 (2) |
| Quaternary | 0 (0) | 5 (0) | 4 (1) | 0 (0) | 2 (1) | 1 (0) |
| Team (# of impacts) |  |  |  |  |  |  |
| Offense | 252 (47) | 631 (49) | 339 (31) | 207 (32) | 231 (36) | 188 (41) |
| Defense | 280 (44) | 514 (40) | 649 (60) | 306 (48) | 302 (48) | 230 (51) |
| Special teams | 49 (8) | 139 (11) | 91 (9) | 128 (20) | 104 (16) | 38 (8) |
| Play type (# of impacts) |  |  |  |  |  |  |
| Pass | 78 (14) | 280 (22) | 226 (21) | 118 (18) | 111 (17) | 76 (17) |
| Run | 454 (78) | 871 (68) | 762 (71) | 395 (62) | 420 (66) | 341 (75) |
| Special teams | 49 (8) | 133 (10) | 91 (8) | 126 (20) | 106 (17) | 39 (8) |
| Unclear | 0 (0) | 0 (0) | 0 (0) | 2 (0) | 0 (0) | 0 (0) |

Unclear: some impact characteristics (i.e., external source, play type) were unable to be derived because they were unviewable (e.g., a heap of players); unviewable characteristics were recorded as unclear.

^a^ N=17 players sustained impacts; n=3 played and did not sustain any impacts

^b^ N=14 players sustained impacts

^c^ N=10 players sustained impacts; n=1 played and did not sustain any impacts

^d^ N=19 players sustained impacts; n=1 played and did not sustain any impacts

^e^ N=11 players sustained impacts; n=2 players were on the team but did not play; n=1 played and did not sustain any impacts

^f^ N=12 players sustained impacts; n=1 player was on the team but did not play
