# Supplementary material for: Longitudinal, prospective study of head impacts in male high school football players
Source: PLoS One. 2023 Sep 8;18(9):e0291374. doi: 10.1371/journal.pone.0291374 (PMC10490840; doi:10.1371/journal.pone.0291374)
Supplement: S2 Table — (DOCX) [file pone.0291374.s003.docx]

**Supplement 3. Impact Characteristics by Position Using Impacts Derived From the HITS Data**

|  | **All Cohorts** | **Cohort 1** | | | **Cohort 2** | | **Cohort 3** |
| --- | --- | --- | --- | --- | --- | --- | --- |
| **Player-level impact characteristics (N=53)** | **All Years**  **(n=53)** | **Year 1**  **(n=20)** | **Year 2**  **(n=14)** | **Year 3**  **(n=11)** | **Year 1**  **(n=20)** | **Year 2**  **(n=14)** | **Year 1**  **(n=13)** |
| Center |  |  |  |  |  |  |  |
| Number of active players | 5 | 3 | 1 | 2 | 1 | -- | 1 |
| Number of plays | 1,515 | 111 | 325 | 407 | 283 | -- | 389 |
| Number of impacts | 431 | 24 | 135 | 120 | 54 | -- | 98 |
| Rate of impacts/play | 0.28 | 0.22 | 0.42 | 0.29 | 0.19 | -- | 0.25 |
| Offensive guard/tackle |  |  |  |  |  |  |  |
| Number of active players | 21 | 10 | 3 | 5 | 6 | 4 | 3 |
| Number of plays | 3,698 | 356 | 768 | 606 | 493 | 757 | 718 |
| Number of impacts | 557 | 61 | 211 | 16 | 73 | 163 | 33 |
| Rate of impacts/play | 0.15 | 0.17 | 0.28 | 0.03 | 0.15 | 0.22 | 0.05 |
| Tight end |  |  |  |  |  |  |  |
| Number of active players | 20 | 5 | 4 | 2 | 7 | 4 | 2 |
| Number of plays | 1,875 | 177 | 490 | 154 | 364 | 337 | 353 |
| Number of impacts | 274 | 13 | 127 | 63 | 25 | 6 | 40 |
| Rate of impacts/play | 0.15 | 0.07 | 0.26 | 0.41 | 0.07 | 0.02 | 0.11 |
| Wide receiver |  |  |  |  |  |  |  |
| Number of active players | 19 | 5 | 5 | 4 | 6 | 5 | 4 |
| Number of plays | 937 | 104 | 208 | 1 | 93 | 253 | 278 |
| Number of impacts | 62 | 5 | 19 | 0 | 6 | 15 | 17 |
| Rate of impacts/play | 0.07 | 0.05 | 0.09 | 0.00 | 0.07 | 0.06 | 0.06 |
| Running back |  |  |  |  |  |  |  |
| Number of active players | 16 | 6 | 7 | 3 | 4 | 5 | 1 |
| Number of plays | 999 | 270 | 270 | 87 | 248 | 118 | 6 |
| Number of impacts | 459 | 125 | 122 | 133 | 32 | 47 | 0 |
| Rate of impacts/play | 0.46 | 0.46 | 0.45 | 1.52 | 0.13 | 0.40 | 0.00 |
| Quarterback |  |  |  |  |  |  |  |
| Number of active players | 5 | 3 | 1 | 1 | 1 | 1 | -- |
| Number of plays | 733 | 151 | 384 | 79 | 86 | 33 | -- |
| Number of impacts | 65 | 24 | 17 | 7 | 17 | 0 | -- |
| Rate of impacts/play | 0.09 | 0.16 | 0.04 | 0.09 | 0.20 | 0.00 | -- |
| Defensive tackle/end |  |  |  |  |  |  |  |
| Number of active players | 30 | 11 | 6 | 8 | 11 | 6 | 6 |
| Number of plays | 4,239 | 398 | 640 | 1064 | 907 | 924 | 306 |
| Number of impacts | 962 | 94 | 141 | 373 | 146 | 129 | 79 |
| Rate of impacts/play | 0.23 | 0.23 | 0.22 | 0.35 | 0.16 | 0.14 | 0.26 |
| Linebacker |  |  |  |  |  |  |  |
| Number of active players | 24 | 10 | 5 | 5 | 7 | 4 | 6 |
| Number of plays | 4,428 | 713 | 880 | 853 | 689 | 910 | 383 |
| Number of impacts | 1,056 | 152 | 305 | 270 | 122 | 136 | 71 |
| Rate of impacts/play | 0.24 | 0.21 | 0.35 | 0.32 | 0.18 | 0.15 | 0.19 |
| Defensive back |  |  |  |  |  |  |  |
| Number of active players | 24 | 7 | 5 | 4 | 10 | 5 | 3 |
| Number of plays | 2,896 | 325 | 536 | 195 | 908 | 522 | 410 |
| Number of impacts | 347 | 34 | 68 | 6 | 122 | 37 | 80 |
| Rate of impacts/play | 0.12 | 0.10 | 0.13 | 0.03 | 0.13 | 0.07 | 0.20 |
| Kicker/punter |  |  |  |  |  |  |  |
| Number of active players | 7 | 2 | 2 | 1 | 1 | 2 | 1 |
| Number of plays | 125 | 18 | 58 | 3 | 3 | 40 | 3 |
| Number of impacts | 7 | 5 | 2 | 0 | 0 | 0 | 0 |
| Rate of impacts/play | 0.06 | 0.28 | 0.03 | 0.00 | 0.00 | 0.00 | 0.00 |
| Kick-punt returner |  |  |  |  |  |  |  |
| Number of active players | 10 | 2 | 2 | -- | 3 | 3 | 1 |
| Number of plays | 97 | 16 | 53 | -- | 5 | 12 | 11 |
| Number of impacts | 24 | 3 | 8 | -- | 4 | 0 | 9 |
| Rate of impacts/play | 0.25 | 0.19 | 0.15 | -- | 0.80 | 0.00 | 0.82 |
| Special teams |  |  |  |  |  |  |  |
| Number of active players | 49 | 19 | 14 | 9 | 17 | 11 | 12 |
| Number of plays | 2,802 | 319 | 678 | 282 | 562 | 577 | 384 |
| Number of impacts | 518 | 41 | 129 | 91 | 124 | 104 | 29 |
| Rate of impacts/play | 0.18 | 0.13 | 0.19 | 0.32 | 0.22 | 0.18 | 0.08 |
